# A Rare Case of Renal Infarct due to Noncompaction Cardiomyopathy: A Case Report and Literature Review

**DOI:** 10.1155/2016/6789149

**Published:** 2016-02-28

**Authors:** Karan Wats, On Chen, Nupur Nippun Uppal, Syeda Atiqa Batul, Norbert Moskovits, Vijay Shetty, Jacob Shani

**Affiliations:** ^1^Internal Medicine, Maimonides Medical Center, Brooklyn, NY 11219, USA; ^2^Department of Cardiology, Maimonides Medical Center, Brooklyn, NY 11219, USA; ^3^Department of Nephrology, North Shore Long Island Jewish Hospital, New Hyde Park, NY 11040, USA

## Abstract

Left ventricular noncompaction cardiomyopathy is a rare myocardial disorder which results from failure of left ventricle to compact in embryogenesis. We present a case of a 53-year-old female who came because of abdominal pain and was found to have renal infarct secondary to noncompaction cardiomyopathy.

## 1. Introduction

Left ventricular noncompaction is a rare form of genetic cardiomyopathy that occurs due to arrest in the compaction of developing myocardium which leads to deep trabeculae and recesses giving the ventricle a spongiform appearance [1–4]. It has been grouped under “genetic cardiomyopathy” by the ACC/AHA [5]. While it most commonly presents as heart failure; ventricular arrhythmias, systemic embolism, or sudden death can occur [4, 6, 7]. While thromboembolic events are not uncommon, they usually present as cardioembolic stroke. We present a case of renal infarct in a middle aged female who presented with abdominal pain and was found to have noncompaction on echocardiography. In our search we found no cases of isolated renal emboli as a presentation of left ventricular noncompaction cardiomyopathy.

## 2. Case Presentation

**Figure 1:**
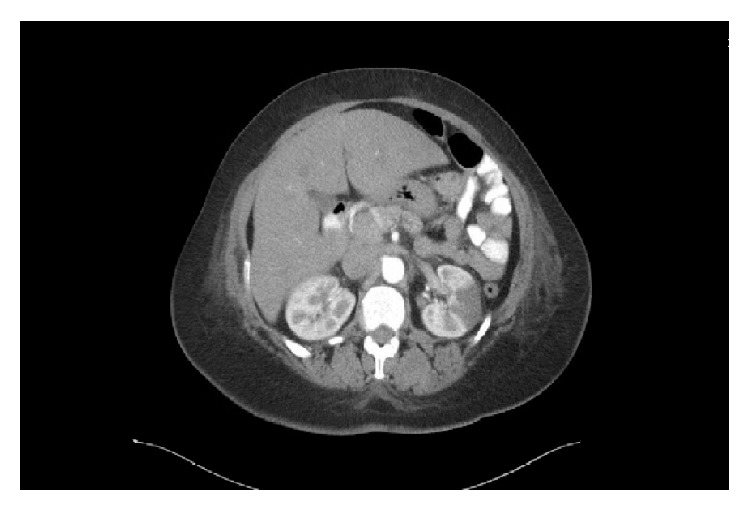
CT scan axial view showing wedge-shaped hypoenhancement in the left kidney marked with an arrow consistent with renal infarct.

**Figure 2:**
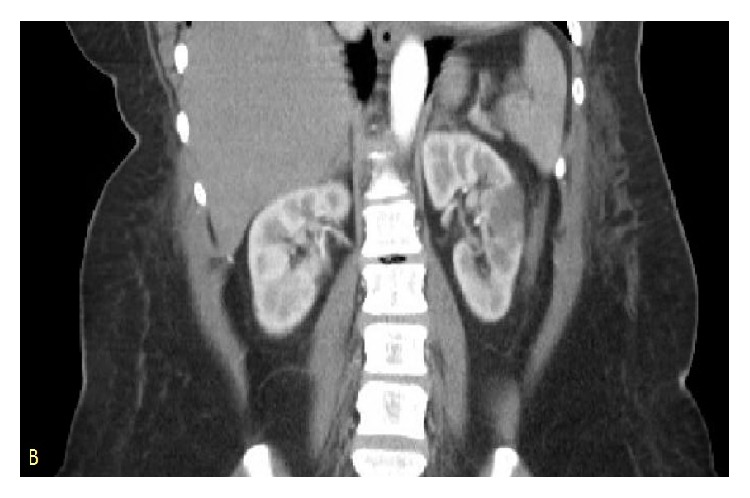
Coronal view of the abdomen showing an area of hypoenhancement marked by an arrow consistent with renal infarct.

A 53-year-old female with a history of hypertension presented with the complaints of left sided back pain and dysuria for 2 days. Patient denied any history of chest pain, shortness of breath, or any cardiac problems in the past. Physical examination was significant for bibasilar crackles and pedal edema but no costovertebral tenderness. There was no family history of cardiomyopathy or heart failure. Routine labs showed hemoglobin, 10.5; white cell count, 6.4; platelets, 391. Renal and liver functions tests were within normal limits. An EKG test was done on admission which showed normal sinus rhythm and left axis deviation but no significant ST-T wave changes. Urine analysis was done which was negative for urinary tract infection. CT scan of abdomen (Figures 1 and 2) showed multiple areas of wedge-shaped hypoenhancement in the left kidney with mild adjacent stranding consistent with renal infarcts.

**Figure 3:**
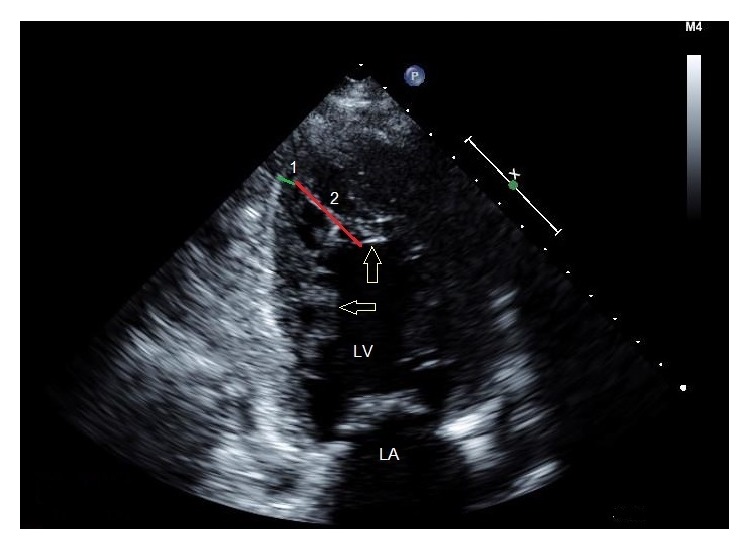
Echocardiographic 2 chamber view showing noncompacted myocardium in the left ventricle marked by yellow arrows. It also shows the ratio of noncompacted/compacted myocardium marked by red and green lines, respectively, with a ratio >2 : 1. LA: left atrium, LV: left ventricle, 1–0.59 cm and 2–2.14 cm as measured.

**Figure 4:**
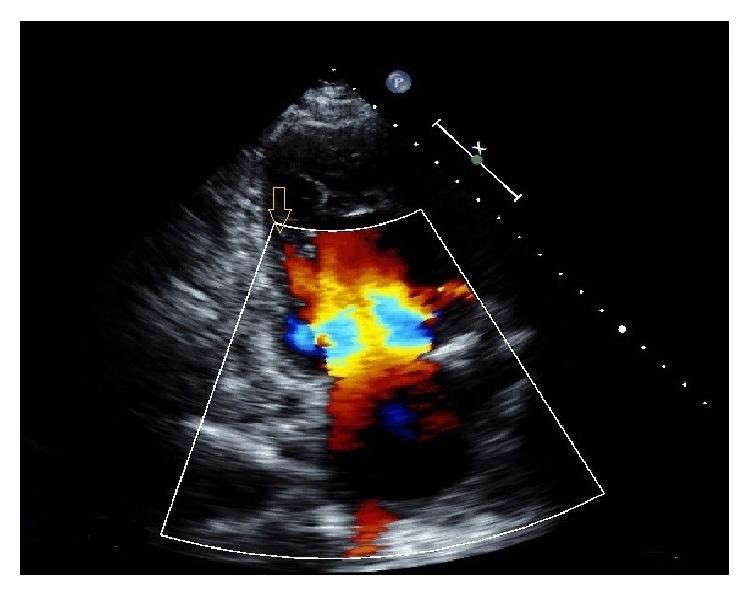
Color Doppler showing blood flow in sinusoids in the left ventricle.

Echocardiogram (Figures 3 and 4) showed a left ventricular ejection fraction, 25% with dilated left ventricle, prominent left ventricular trabeculations, and deep intertrabecular recesses communicating with the LV cavity, consistent with left ventricular noncompaction. Patient underwent an elective angiogram which revealed normal coronaries. Patient was started on heparin for renal emboli and bridged to Coumadin. She was discharged to home with a cardiologist follow-up and INR checks.

## 3. Discussion

Left ventricular noncompaction (LVNC) was first described in 1975 by Dusek et al. [1] in infant hearts as spongy myocardium. It was later described in adults by Engberding and Bender [2] as lack of sinusoidal regression in embryogenesis. Isolated left ventricular noncompaction is a rare form of cardiomyopathy which has been grouped under the genetic cardiomyopathy by the American Heart Association which is caused by arrest of compaction of myocardial fibers during embryogenesis [2–5]. It is characterized by (1) an abnormal myocardial structure characterized by prominent trabeculae and deep intertrabecular recesses with two layers of myocardium, a compacted epicardial layer and a noncompacted endocardium. (2) There is continuity and blood flow from LV to these deep intertrabecular recesses which are filled with blood but there is no evidence of communication with the epicardial coronary artery system [8, 9].

LVNC is a genetically heterogeneous disorder and both familial and sporadic forms have been described. Mutations in various genes [10–12] have been described. Familial forms have been associated with mutations in mitochondrial, sarcomeric, and cytoskeletal genes. A study conducted by Murphy et al. [13] found 8 affected family members among 32 patients. Although genetic testing is not routinely recommended at this time, the Heart Failure Society of America practice guidelines [14] recommend clinical screening of all first-degree relatives of affected patients for LVNC.

The incidence of isolated noncompaction varies among different studies but has been noted to be anywhere between 0.014 and 1.3%. A lot of patients with LVNC are diagnosed during the neonatal period. A significant number of these patients have undulating phenotype in which they have transient recovery of function and present with symptoms later in adult life. Most of the times in children, LVNC usually coexists with other genetic conditions like Barth syndrome, Emery-Dreifuss muscular dystrophy, myotubular myopathy, and so forth, in contrast to adults where it has been known to occur in isolation. It is still unclear whether presentation of LVNC in adulthood represents a long standing condition or delayed manifestation of molecular pathology. With increasing awareness of this rare condition and better and more specific echocardiographic diagnostic parameters, the prevalence of this condition has increased.

The most common forms of presentation include heart failure, arrhythmias, and systemic embolism [4, 6, 7]. In a study of 34 adult patients conducted by Oechslin et al. [4], heart failure was the presenting symptom in 68% of patients and arrhythmias most commonly observed included nonsustained and sustained ventricular tachycardia in 41% of patients. Systemic embolism, most commonly cardioembolic stroke, was observed in 21% of patients. Other forms of embolic complication which have been observed include transient ischemic attack, pulmonary embolism, and mesenteric ischemia [8]. Our patient presented with complaints of abdominal and was eventually found to have a renal infarct which is not a common presentation for this rare disease entity.

Echocardiography with color follow Doppler has been considered as the diagnostic modality of choice for diagnosis of this rare cardiomyopathy and specific criteria for diagnosis have been put forward by various researchers [6, 15–18]. Jenni et al. proposed the following criteria for diagnosis of this condition which have been shown to be highly specific for this condition:

1. A thickened ventricular wall consisting of two layers, a thin compacted epicardial layer and a markedly thickened endocardial layer with numerous trabeculations and deep recesses with a maximum ratio of noncompacted to compacted myocardium of >2 : 1 at the end systole in the parasternal short axis view.
2. Color Doppler evidence of flow within the deep intertrabecular recesses.
3. Prominent trabecular meshwork in the LV apex or midventricular segments of inferior and lateral wall

Our patient had an echocardiogram which showed a noncompacted to compacted myocardium ratio of >2 : 1 at the ventricular apex prominent trabecular meshwork in the LV apex and color Doppler evidence of flow in the recesses thus making a diagnosis of LVNC.

Cardiac magnetic resonance imaging (Cardiac MRI) has been increasingly used for diagnosis of noncompaction cardiomyopathy as it provides a detailed image of cardiac morphology. Cardiac MRI is particularly useful in patients in whom the apex is difficult to visualize with echocardiography or in whom the diagnosis is uncertain with conventional echocardiography. A noncompacted/compacted ratio >2.3 measured at end diastole is used as the diagnostic indicator for noncompaction cardiomyopathy [19]. A study comparing the use of echocardiography versus Cardiac MRI showed no significant difference at the end diastole but Cardiac MRI at end systole was able to better characterize the extent of compaction [20]. Despite this small difference, echocardiography continues to be the diagnostic modality of choice for noncompaction cardiomyopathy.

Symptoms of heart failure are the most commonly presenting symptoms in LVNC. Tian et al. [21] found in their retrospective review that 60% with LVNC were in New York Heart Association (NYHA) functional class III/IV and 79% had systolic dysfunction (left ventricular ejection fraction (LVEF) <50%). The treatment principles remain the same as any other patient presenting with heart failure. Conventional guidelines for management of heart failure by ACC/AHA should be used when managing patients with LVNC. Patients with symptoms despite optimal medical management should be considered for implantable cardioverter defibrillator (ICD) implantation.

Sudden cardiac death is another concern in these patients and should always be evaluated for presence of sustained/nonsustained ventricular tachycardia. The incidence of ventricular arrhythmias varies from 6% [22] to 62% [6] and can lead to sudden death. ICD therapy may be considered in such cases as measure of primary prevention and should also be used in patients who have sustained ventricular tachycardia or cardiac arrest for secondary prevention. According to the Device Based Therapy guidelines [23], implantation of ICD for the prevention of sudden death in patients with LVNC is a class IIb recommendation.

Systemic embolism is another complication associated with LVNC. Initially it was thought to result from stagnation of flow across the prominent trabeculations and deep recesses leading to clot formation in noncompacted layer. A retrospective review done by Stöllberger and Finsterer [24] found that the rate of clot formation and embolism in LVNC is related to the presence of concomitant conditions like low EF, presence of atrial fibrillation, or both rather than LVNC alone. Therefore, oral anticoagulation is recommended in patients with concomitant low EF, atrial fibrillation, history of systemic embolism, and so forth.

Studies on asymptomatic patients with normal EF failed to show any increased risk of systemic embolism and therefore use of anticoagulation in this patient population remains controversial [25].

Large case series done by Lofiego et al. [22] and Greutmann et al. [26] for outcomes in patients with isolated LV noncompaction has shown that the prognosis differed depending on the presence or absence of symptoms at presentation. During follow-up of 46 months and 32 months in these case series, respectively, cardiovascular death and heart transplantation needed to be done in 31% of the patients as compared to none in any of the asymptomatic patients.

A recent retrospective review done by Tian et al. [21] in 106 patients with LVNC showed that 28 (26%) patients died or underwent heart transplantation during a follow-up of 2.9 ± 2.1 years. The study also showed that advanced heart failure, a dilated left heart with systolic dysfunction, reduced systolic blood pressure, pulmonary hypertension, and right bundle branch block predict adverse outcomes of LVNC at the time of diagnosis.

Another recent study [27] done on pediatric population showed that children who had normal or mild left ventricular dysfunction had much better prognosis in terms of arrhythmia burden and sudden cardiac death incidence further strengthening the importance of left ventricular function at the time of diagnosis and its impact on future health and outcomes.

## 4. Conclusion

While LVNC is not common, its prevalence has increased due to better imaging modalities and more specific diagnostic criteria but still this disease may be underdiagnosed due to lack of awareness among clinicians. Our case illustrates an uncommon presentation of LVNC with renal infarction as the presenting manifestation which later leads to finding of severely decreased LV function and diagnosis of LVNC. It is important to recognize that this condition as the management of LVNC is slightly different from other causes of LV dysfunction such as the need to anticoagulate because of the high risk of thromboembolism and the familial inheritance requiring genetic counselling, screening among family members.

This makes the disease, its presentations, complications, and management important for the general internist and the cardiologist to know.

## Supplementary Material

Echocardiographic apical two chamber view showing the non-compacted myocardium most prominent at the apex.

## Abbreviations

ACC: American College of Cardiology
AHA: American Heart Association
LV: Left ventricle
EF: Ejection fraction
LVNC: Left ventricular noncompaction
ICD: Implantable cardioverter defibrillator
MRI: Magnetic resonance imaging
NYHA: New York Heart Association.
